# Morphology and Nanomechanics of Sensory Neurons Growth Cones following Peripheral Nerve Injury

**DOI:** 10.1371/journal.pone.0056286

**Published:** 2013-02-13

**Authors:** Marta Martin, Ouafa Benzina, Vivien Szabo, Attila-Gergely Végh, Olivier Lucas, Thierry Cloitre, Frédérique Scamps, Csilla Gergely

**Affiliations:** 1 Université Montpellier 2, Laboratoire Charles Coulomb UMR 5221, Montpellier, France; 2 CNRS, Laboratoire Charles Coulomb UMR 5221, Montpellier, France; 3 Laboratoire LVBPPE- Centre de Biotechnologie de Sfax- BP 1177, Sfax, Tunisie; 4 Institute of Biophysics, Biological Research Centre of the Hungarian Academy of Sciences, Szeged, Hungary; 5 INSERM U 1051 INM-Hôpital St Eloi, Montpellier, France; Clarkson University, United States of America

## Abstract

A prior peripheral nerve injury *in vivo*, promotes a rapid elongated mode of sensory neurons neurite regrowth *in vitro*. This *in vitro* model of conditioned axotomy allows analysis of the cellular and molecular mechanisms leading to an improved neurite re-growth. Our differential interference contrast microscopy and immunocytochemistry results show that conditioned axotomy, induced by sciatic nerve injury, did not increase somatic size of adult lumbar sensory neurons from mice dorsal root ganglia sensory neurons but promoted the appearance of larger neurites and growth cones. Using atomic force microscopy on live neurons, we investigated whether membrane mechanical properties of growth cones of axotomized neurons were modified following sciatic nerve injury. Our data revealed that neurons having a regenerative growth were characterized by softer growth cones, compared to control neurons. The increase of the growth cone membrane elasticity suggests a modification in the ratio and the inner framework of the main structural proteins.

## Introduction

The dorsal root ganglia contain a variety of sensory neurons that transduce somatic stimuli [1]. Following peripheral nerve injury, sensory neurons have to adapt to a new environment in order to successfully promote their axonal elongation. Unsuccessful regeneration leads to post-traumatic neuropathies, such ataxia and pain-related behaviour, which are often chronic and mostly resistant to current treatments. Understanding the cellular and molecular mechanisms leading to improved neurite re-growth is a major step to propose new therapy for nerve repair. To this end, numerous studies analysed transcriptional [2]–[5] and post-transcriptional modifications [6], [7] induced by nerve injury at the somatic and axonal level. Among the cellular mechanisms leading to improved neurite growth, it has been demonstrated that a prior *in vivo* nerve injury enhances both peripheral and central axonal regeneration following a second injury [8], [9]. *In vitro*, the neurons conditioned by the first traumatism display a faster, elongated mode of neurite growth called regenerative growth [10], [11]. This provides a convenient model to decipher post-transcriptional molecular adaptation involved in regenerative growth [12], [13].

Moreover, in addition to analysis of biochemical processes, this model allows assessing physical modifications of neuronal membrane properties that could also contribute to regenerative neurite growth. The neuronal growth cone is a highly motile mechanosensitive structure at the tip of the axon. It receives multiple extracellular guidance cues through surface receptors and transduces this information into directional movements [14], [15]. Numerous studies have uncovered the various guidance molecules and underlying signalling pathways [16], [17] as well as the role of F-actin and microtubule cytoskeleton in growth cone movements [18]. Elasticity is a determining parameter of membrane mechanical properties and provides important information toward the health and function of the cell [19]. There is increasing evidence that membrane tension influences growth cones dynamics [20]. However, knowledge about the three-dimensional organization and rheological properties, such as elasticity, of distinct growth cone regions under live conditions has remained sparse [21]. To date, no data are available on the effects of conditioning injury on the nanoscaled morphology and mechanical properties of growth cone membranes of sensory neurons.

Cellular elasticity measurements have been made using techniques such as micropipette aspiration [22], the biomembrane force probe [23], optical tweezers [24], [25] and magnetic tweezers [26]. Atomic force microscopy (AFM) has become nowadays a common tool for high resolution imaging of biological materials, as it does not require complicated sample preparation and live cells can be imaged in physiological conditions. AFM allows the study of neurite growth cones topography in real time with nanometer resolution and measuring surface forces and surface properties (hydrophobicity, elasticity) at the nanoscale providing spatially–resolved maps of the nanomechanical characteristics of growth cones. AFM has been used to measure elasticity of many cellular types [27], [28]. However, very few experiments involved neurons [29]–[32] particularly in the living state [33]–[35]. In the present manuscript, we investigated, using AFM, the morphology and the membrane mechanical properties of growth cones of adult sensory neurons from mice dorsal root ganglia (DRG) following left sciatic nerve injury. We demonstrate that under regenerative growth, growth cones are much softer than those from control neurons, as they have a lower Young's modulus. Morphological analysis of growth cones supports that change in growth cone membrane elasticity correlates with modification in the ratio and structure of the main structural proteins, actin and βIII-tubulin. These studies have been completed with the investigation of neurite outgrowth via differential interference contrast microscopy and immunocytochemistry.

## Results

### Morphological characterization via optical microscopy

**Figure 1:**
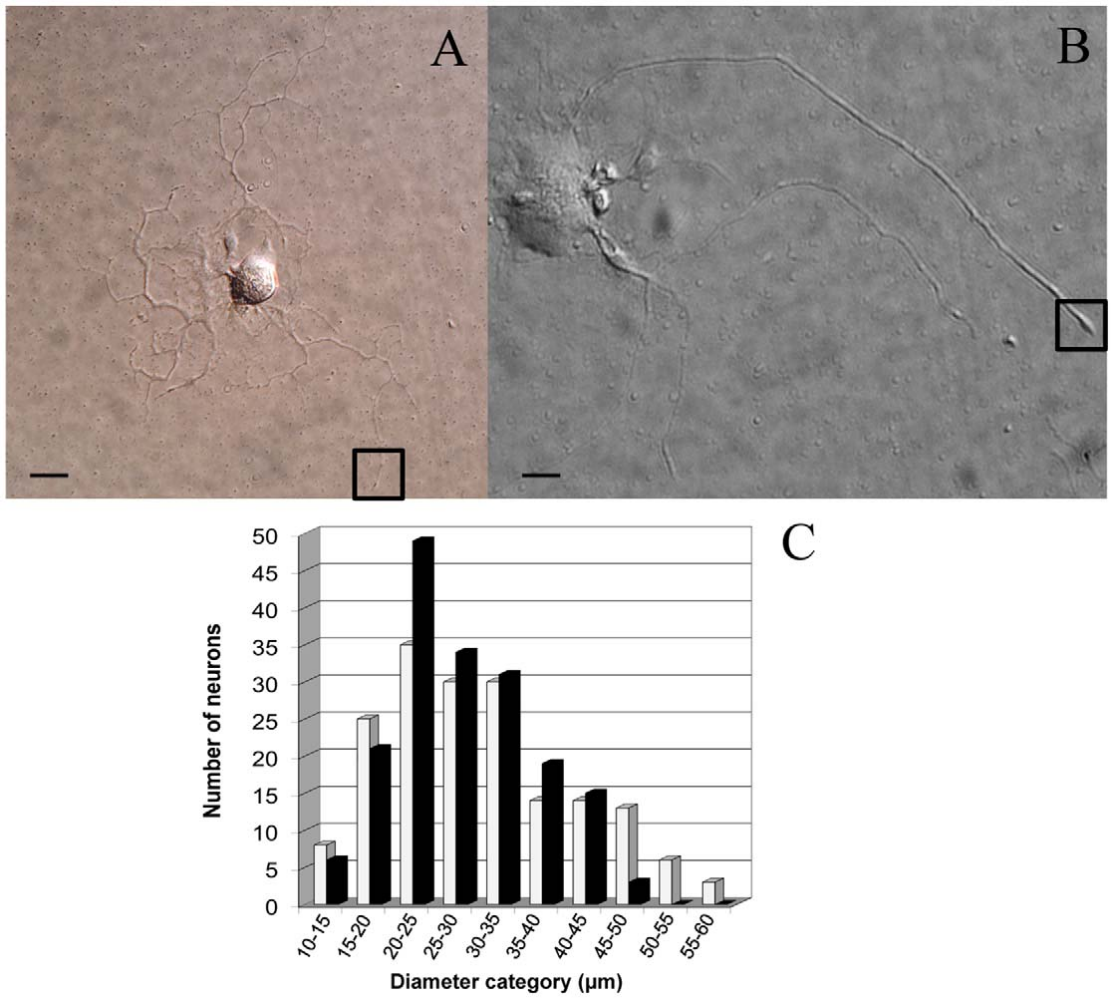
Differential interference contrast microscopy images of mice dorsal root ganglion (DRG) sensory neurons, revealing a clear three-dimensional appearance. (A) DIC image of an arborizing control neuron (B) DIC image of an elongated neural cell following peripheral nerve injury. The boxes depict two growth cone regions suitable for AFM investigations. (Scale bar 20 µm). (C) Size-distribution of cultured neurons after 24 hours as measured in phase contrast microscopy. The histogram shows no significant difference between the somatic diameters of normal (white bars) and lesion-conditioned neurons (black bars) between 10–45 µm diameters. Axotomy induced the disappearance of large sized somatic diameters (45–60 µm).

After one day in vitro (1DIV), prior *in vivo* nerve injury induced neurite outgrowth among conditioned axotomized sensory neuron cultures with a clearly different morphology compared with control neuron cultures. As previously reported [11], most control neurons displayed an arborizing neurite growth characterized by numerous branching (Fig. 1A), whereas conditioned axotomized sensory neurons presented an elongated neurite growth characterized by significantly less branching, longer neurite length and the appearance of large growth cones (Fig. 1B). To further assess morphological modifications between control and lesion-conditioned neurons, somatic diameters were measured at 1DIV using phase contrast microscopy. In control neurons, cell somatic diameter amounted 30.9±8.8 µm, n = 179 (ranged from 10 µm to 60 µm). Somatic diameter of conditioned neurons was significantly less amounting 28.8±6.8 µm, n = 179 (p = 0.04, t-test and ranged from 10 µm to 50 µm) (Fig. 1C). The frequency distribution histogram evidenced that axotomy mainly induced the disappearance of the neuronal population having large somatic diameter (i.e. 50–60 µm). Under both condition, the majority of neurons had a somatic size comprised between 20 µm and 30 µm and neurons in this range size were selected for AFM experiments.

**Figure 2:**
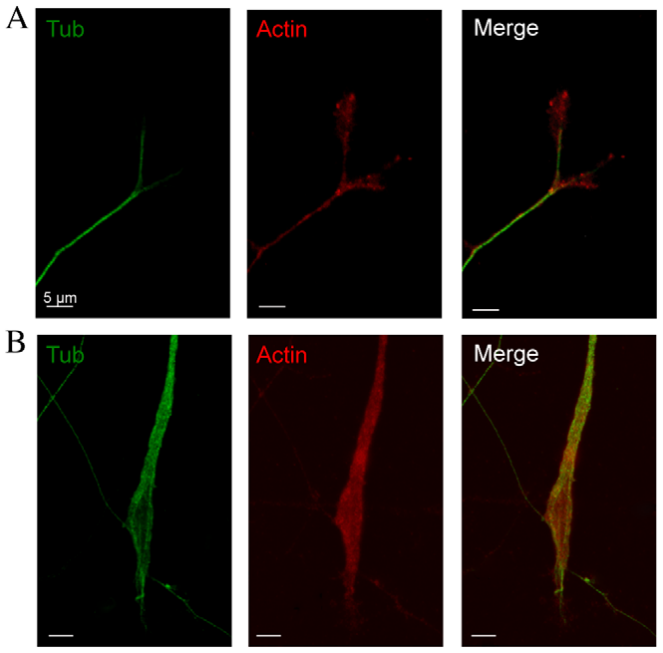
Immunostaining of βIII-tubulin and actin in growth cones from control and axotomized sensory neurons. (A) Confocal images of double immunostaining with anti- βIII-tubulin (Tub, green) and anti-actin antibodies (Actin, red) in control sensory neuron at 1DIV. Merged image shows preferential actin localization to growth cone tip. (B) Confocal images of double immunostaining with anti- βIII-tubulin and anti-actin antibody in axotomized sensory neuron at 1DIV. Neurite and growth cone are larger than in control and merged image shows a slight actin spreading as observed in control in combination with a very thin tubulin prolongation at the growth cone tip. (Scale bars 5 µm, ×63).

Nerve injury also promoted the appearance of larger neurites and growth cones compared to control neurons, as illustrated in Fig. 1B. To evidence structural differences between conditioned and control growth cones, we analysed actin and βIII-tubulin, known growth cone cytoskeletal components. Actin is expressed in lamellipodium and filopodium composing the growth tip, while microtubules are structural support for axon elongation and substrates for fast axonal transport of organelles into the growth cone [18]. Therefore, we performed a morphological analysis using immunocytochemistry to localize actin (anti-actin antibody) and neuronal microtubule (anti-βIII-tubulin). In agreement with DIC images shown in Fig. 1B, growth cones of conditioned neurons are larger with the form of a membrane swelling composed of both actin and βIII-tubulin (Fig. 2). At the tip of the growth cone, actin is hardly visible in conditioned neurons (Fig. 2B) as compared with a clear spreading of actin in control neurons (Fig. 2A).

### Growth cones morphology using AFM

**Figure 3:**
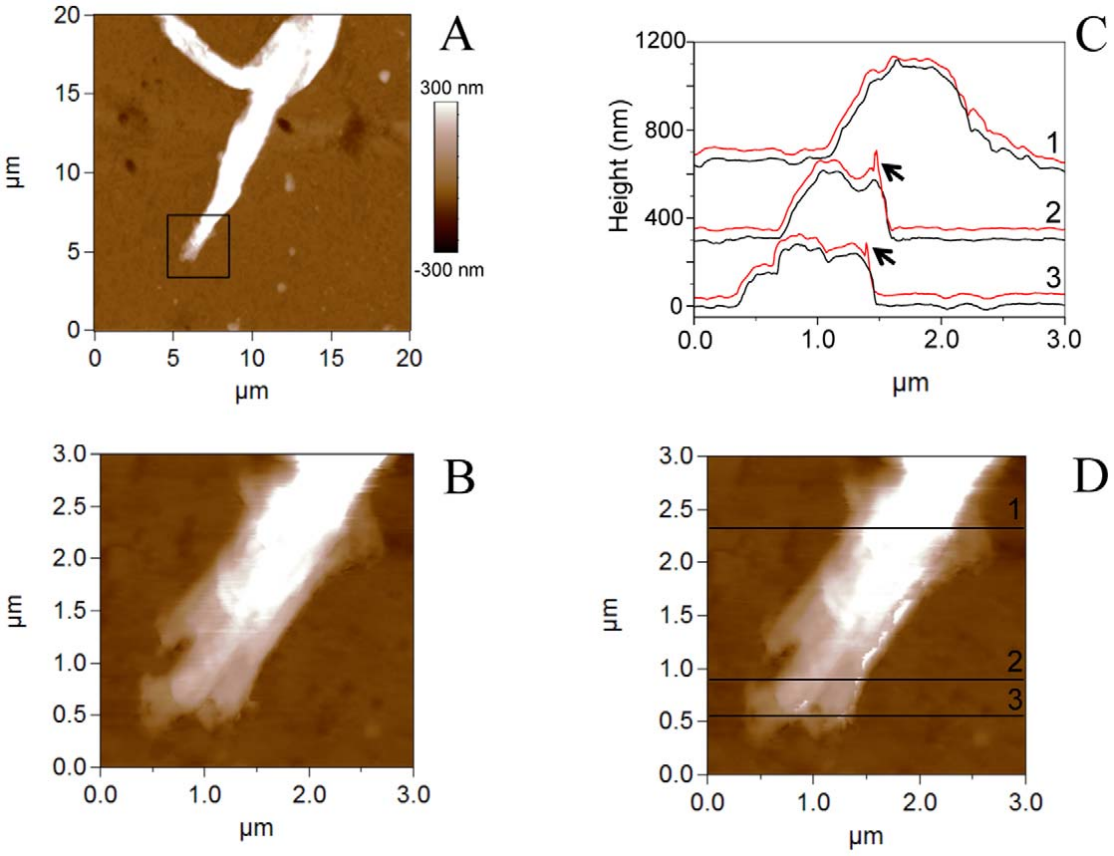
AFM images in contact mode of a growth cone from conditioned axotomized sensory neuron. (A) Height AFM image of the growth cone region. The box represents the area scanned in (B) trace image (scanning direction from left to right) and (D) retrace image (scanning direction from right to left). (C) Height profiles of the cross sections indicated in (D) corresponding to the trace (black lines) and the retrace images (red lines). For all profiles, both lines show similar structures suggesting that the imaging force did not perturb the growth cone integrity. Black arrows indicate the folding of the membrane.

The morphology of live growth cone was further studied using AFM. Typical AFM images taken in contact mode of a growth cone from a conditioned sensory neuron are shown in figure 3. AFM contact mode gives the height image of the growth cone region (Fig. 3A) and to ensure that contact mode did not affect growth cone structure, the trace and the retrace images were scanned (Fig. 3B–D). The structures shown in both images are very similar, suggesting that the force imaging did not trouble the cone growth integrity. Although the height profiles of the cross sections in the retrace image are very consistent with the profiles of the trace image, we observe the folding of some portions of the membrane when the scanning direction is from right to left (Fig. 3C).

**Figure 4:**
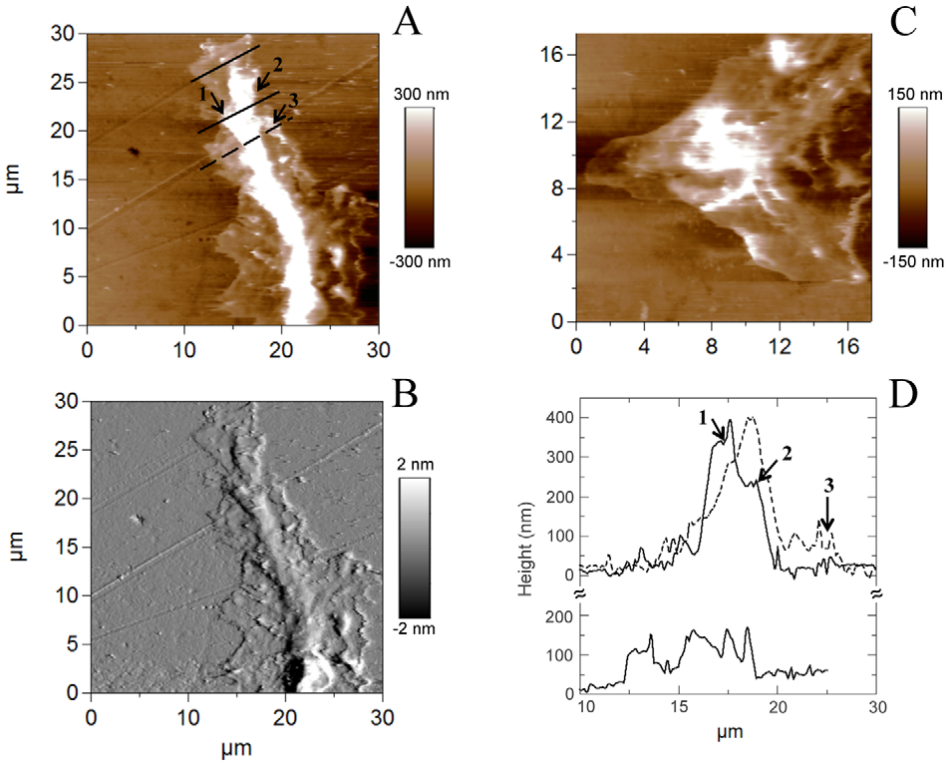
AFM images in contact mode of control growth cones. (A), (C) Topography of two different growth cones; (B) Corresponding deflection image of A and (D) Cross sections along the lines indicated in A. Arrows indicate areas with different heights ranging from 100 nm (area 3) to 350 nm (area 1), and located in the P and C domains, respectively.

Three main domains can be distinguished in the growth cones [21] amongst which two can be well resolved by AFM: the thicker part (called C domain) located closest to the cell body containing mainly stable microtubules and a flat (P) domain at the edge of the growth cone composed primarily of dense actin meshwork and bundled F-actin and where highly dynamic microtubules extend to explore the periphery. In agreement with the presence of two AFM resolvable domains, our AFM images of growth cones of control neurones show that the growth cone tip is constituted by a rather thin flat structure (P domain, range 0–100 nm) surrounding a main higher structure (C domain, 200–400 nm) (Fig. 4). On both growth cones “veil-like” structures can be well resolved adjacent to the leading edge of the growth cone corresponding to the lamellipodia.

**Figure 5:**
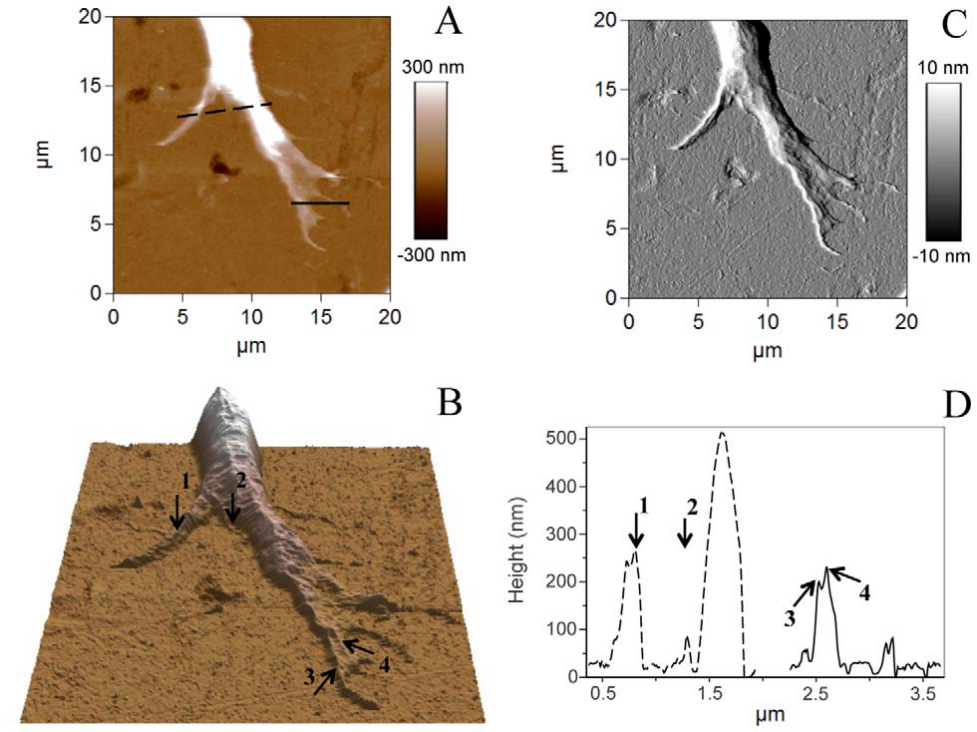
AFM image in contact mode of a conditioned axotomized growth cone. (A) Topography; (B) 3-D reconstruction; (C) Corresponding deflection image and (D) Cross sections along the two lines indicated in B, corresponding to the P and C domains. Arrows indicate several elevated structural features and their corresponding height in the section profile (D).

We next recorded growth cones from conditioned axotomized sensory neurons. We have always imaged the most isolated growth cone within the neuron branched structure. Figure 5 illustrates height and deflection images as well as a 3-D reconstruction and height profiles of an axotomized growth cone. As in control neurons, both flat (P domain, 100–200 nm) and elevated (C domain, about 500 nm) structures could be observed at the growth cone tip (Fig. 5B, D and Fig. 3). We also observe the veiled lamellipodia and fine extensions at the edge of the growth cone corresponding to the filopodia of the P domain. Similar well resolved images have been previously reported for *Aplysia* (non-vertebrate sea-slug) growth cones that are 5–10 fold larger than the ones studied by us [21]. High resolution AFM images on living chick embryo dorsal root ganglion have been reported [30], [33], [34]. However, our AFM images constitute the first high resolution pictures of injury conditioned living vertebrate sensory neuron growth cones revealing their structural details.

### Growth cones nanomechanics measured by AFM

**Figure 6:**
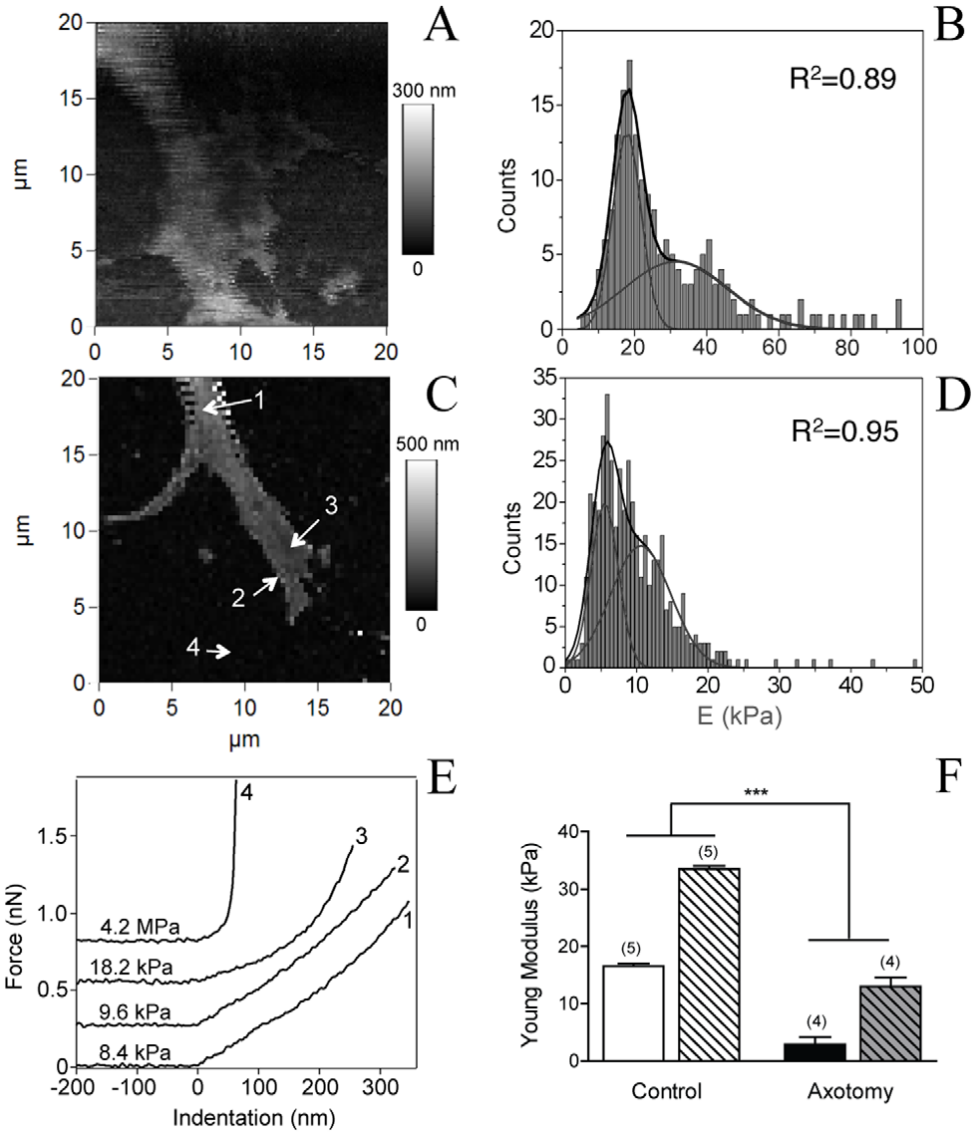
AFM force-volume maps of growth cones indicating the stiffness variation: (A) Elasticity map of a control growth cone and (B) the associated Young's modulus E. Histogram was best fitted with two Gaussians giving mean values of 17.9±0.6 kPa and 34.2±2.3 kPa. (C) Elasticity map of the conditioned growth cone showed in fig. 5. (D) Corresponding histogram of the associated Young's modulus E, showing mean values of 5.5±0.1 kPa and 10.6±0.4 kPa is also depicted. Arrows indicate several structural features in terms of elasticity. Corresponding forces vs indentation curves are represented (E), indicating that the C domain (force 1) and some features at the extremity of the growth cone (force 2) are softer than the P domain (force 3). Nevertheless, the three forces applied to the growth cone yield to Young's modulus values much lower (kPa) than the forces performed on the neuron-free substrate surface (MPa). (F) Both Young's modulus mean values are significantly smaller in conditioned growth cones compared with the control growth cones (***, p<0.001, t-tests. Number of experiments in bracket).

To follow the effects of conditioning injury on the nanomechanical properties of the live growth cone of sensory neurons, we performed nanoindentation measurements by AFM. These experiments monitor the elasticity of the cell that is related mainly to the intrinsic properties of the cell membrane and cystoskeleton structures such as microtubules, and actin fibers. Forces curves were measured on the growth cone region to investigate the membrane elasticity in both elevated (C) and flat (P) domains. For both types of neurons force-volume images constructed from force curves collected at each point in a two-dimensional scan were acquired in relative triggering mode as 64×64 or 96×96 array force plots. The number of collected pixels is largely sufficient to identify the stiffness variability over the growth cone surface, as well as the topography. A comparison between the AFM force-volume maps of control and conditioned growth cones is shown Figure 6 A and C. Under both conditions, Young's modulus distributions were best fitted with two Gaussians (Fig. 6B, D), suggesting the co-existence of two different elasticity populations that could be correlated to the C-domain (softer Young's modulus values) and to the P-domain (harder values). Values of one-Gaussian fit correlation coefficients (R) were always lower than the corresponding two-Gaussian ones. Our results show a clear decrease in the Young modulus value of both fit components for the growth cones of lesion-conditioned neurons compared to the control ones (Fig. 6F). The average Young's modulus values of the growth cone of sensory neurons after peripheral axotomy were 2.9±0.3 kPa and 13.0±1.5 kPa, n = 4, for the first and the second Gaussian fit component, respectively. These values are significantly lower than those measured for the control neurons: 16.5±0.4 kPa and 33.5±0.8 kPa, n = 5, (p<0.001, t-test) revealing the softening of the growth cone as a whole after conditioning injury. Moreover, after axotomy, the Young's modulus of the P domain becomes six fold harder than of the C domain, in contrast to control growth cones where the difference between them is twofold. The Gaussian distribution with lower Young modulus values demonstrates that the C domain is softer than the P domain. Furthermore, Fig. 6C displays the presence of softer structural features (for example arrow 2), revealing higher elasticity than the surrounding stiffer areas within the P domain. This is well illustrated by the corresponding force versus indentation curves in Fig. 6E. Forces 1 and 2 corresponding to the C domain and the edge of the P domain indicate softer features than the main area of P domain (force 3). Thus, forces 1 and 2 belong to the softer elasticity population and force 3 is located within the second stiffer distribution. Similar differences have been observed previously that can be correlated with the underlying cytoskeletal structure [21]: the P domain, rich in F-actin bundles and actin network, is a stiffer structure than the C domain containing microtubule fibres. Surprisingly, in the lesion-conditioned neurons we observed the presence of softer features also in the P domain, suggesting outgrowth of dynamic microtubules towards the very-end of the cone growth.

**Figure 7:**
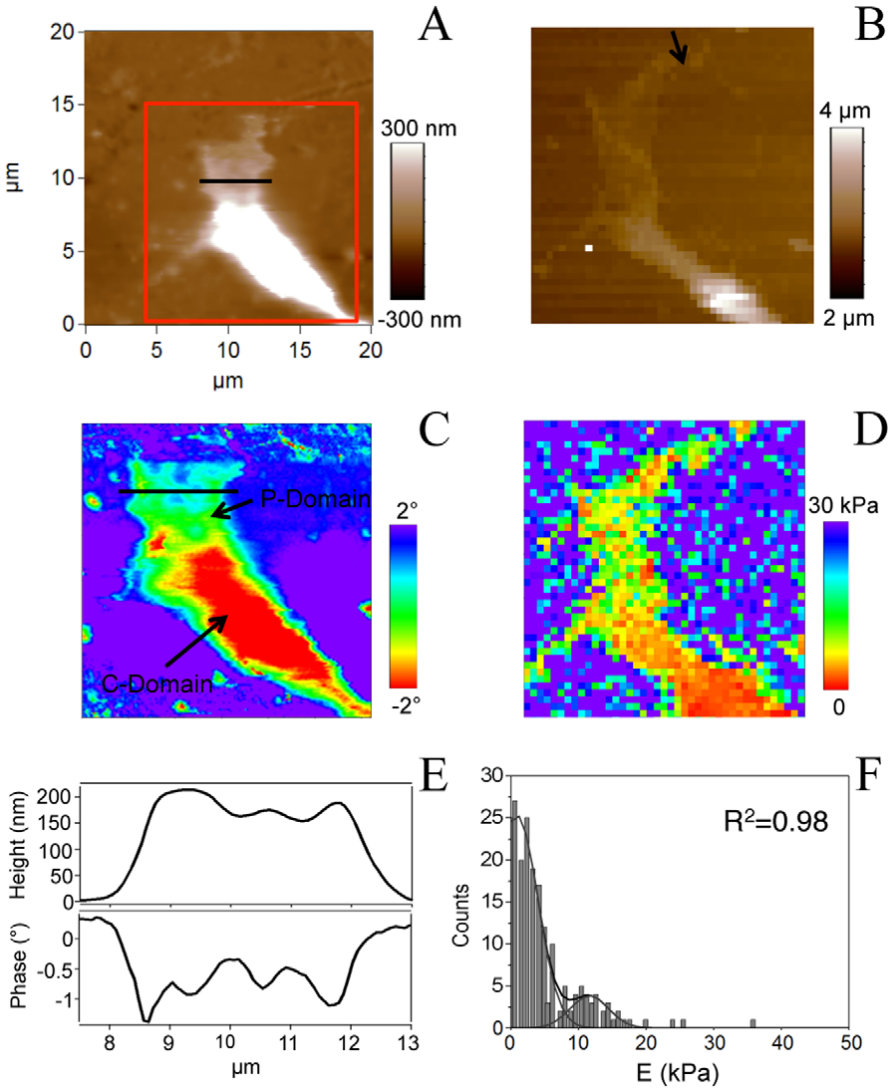
AFM images in tapping mode of a conditioned growth cone. (A) Height AFM image and (C) phase image recorded within the box depicted in A. (B) Height of the contact point recorded during the force-volume measurement, for 64×64 points. This image, measured within 20 minutes after the height image (A), shows several growth cone morphological changes, in the C-domain width and in the P-domain shape. A clear elongation of the tip of the cone to the right of the image (see arrow) becomes visible. (D) Young's Modulus map, showing that the C domain is softer than the P domain. Nevertheless, within the P domain there are softer features. (E) Height and phase profiles along the lines indicated in A and B. The features depicted in the phase section indicate changes in the P-domain properties. (F) Histogram of the associated Young's modulus E showing two Gaussian distributions with mean values of 0.9±0.3 kPa and 11.6±0.7 kPa.

Finally, we used AFM in tapping mode to map simultaneously the shape and the compositional variations of the lesion-conditioned growth cone. Compositional variations are measured by the phase lag of the vibrating probe with respect to the external excitation (phase imaging) [36]. Thus, phase lag of the oscillation enables to explore tip–surface elastic and inelastic interactions. Indeed, it gives a measure of the energy dissipation involved in the contact between the tip and the sample, which depends on a number of factors, including viscoelasticity and adhesion. Fig. 7 shows AFM height (A) and phase images (C) of a lesion-conditioned growth cone. Phase imaging enables compositional variations to be detected, which are otherwise unnoticeable in the topography image, and therefore reveals the inner structure of the growth cone (P and C domains). Considerable differences were observed in the growth cone in terms of sample properties due to variations in composition, hardness, adhesion, or viscoelasticity, also reflected by the height and phase profiles shown in Fig. 7E. Although the height profile is rather flat, the features depicted in the phase section evidence the compositional variations within the P-domain. Nevertheless, there is no clear correlation of height and phase signals (Fig. 7E.). We did not observed remarkable features in C-domain phase profiles (not shown). Panel 7B represents the height of the contact point map recorded during the force-volume measurement. This image, recorded within 20 minutes after the height image (panel A), shows several growth cone morphological changes, in particular the C-domain width and the P-domain shape. Moreover, a clear elongation of the tip of the cone (indicated by an arrow) becomes visible, revealing the evolution of the growth cone with time.

In addition, the image of the calculated Young's modulus (Fig. 7D) confirms that the C domain is softer than the P domain, and that within the P domain there are different regions in terms of elasticity. The histogram of the associated Young's modulus, E, showing two Gaussian distributions is depicted in Fig. 7F.

## Discussion

To our knowledge, our study is the first that investigated the effects of conditioning injury on both morphology and mechanical properties of live sensory neurons growth cones. We demonstrate with AFM that the regenerative neurite growth is characterized by an increase in growth cone elasticity. The reduction in actin content at the growth cone tip together with the increase in βIII-tubulin could account for the changes in membrane biophysical properties.

A prior in vivo peripheral nerve lesion induces an increase in the neurite length in vitro following a second axotomy due to faster rates of outgrowth, straighter neurites with reduced branching frequency [10], [12], [37]. In previous works two possible mechanisms were suggested to explain this so-called regenerative mode of growth: (i) the initially formed side branches are retracted over time as their structural material is incorporated in the main branch and lesioned neurons can faster retract side branches than control neurons (ii) severing neuronal contact with peripheral trophic factor sources can also reduce branching frequency [37].

Growth cones are composed of two main domains, the C and P domains. We confirmed that under control conditions, βIII-tubulin is a major component of the C domain including neurites and branching, while actin is a marker of the P domain involved in lamellipodium and filipodium formation responsible for cone spreading [21], [38]. Growth cones of conditioned sensory neurons display a quite different repartition in skeletal markers with an increase in βIII-tubulin staining and a strong decrease in actin spreading structure reflecting smaller extension of lamellipodium and filipodium.

Using AFM on live sensory neurons, determination of the heights of distinct growth cone regions on live sensory neurons did confirm the P domain as the thinnest growth cone region [21] both in control and conditioned neurons. However, within the P domain of conditioned growth cones, thin and higher features were observed. This observation is quite in agreement with immunocytochemistry results showing a combination of actin network with thin tubulin prolongation structures at the growth cone tip of conditioned neurons. Combining fluorescence microscopy and AFM information enables investigation of cytoskeleton involvement in cellular morphology and mechanics. Though multiple labelling cytoskeleton components (in our case actin and tubulin) involve fixed cells, and AFM advantageously probes live cells, the two kinds of data can be compared if the experiments are done on cells at the same stage (one day *in vitro* following identical neuronal isolation procedure). Similar comparative AFM-fluorescence microscope study has been recently published on live/fixed neurons [35].

Furthermore, our force measurements enabled to investigate the mechanical properties of the neuron growth cones and to probe the structure of the cytoskeleton components underlying the cellular membrane. Our results suggest a correlation between elastic modulus and actin content and organization in distinct growth cone regions. Indeed, in control growth cones, Young's moduli were best fitted with two Gaussian corresponding to the elevated, softer structure in the central C domain and a thinner, twice harder structure in the peripheral, P domain. In agreement with our results, two values in Young moduli have been observed in *Aplysia* growth cones and attributed to actin bundles in the P domain (10–40 kPa), and the softer microtubules in the C domain (3–7 kPa), respectively [21]. Correlation with the main structural proteins, actin and βIII-tubulin supports that actin confers a hard structure while βIII-tubulin is responsible for softer material. Following nerve injury, we evidenced a decrease in the Young's moduli of growth cones of lesion-conditioned neurons compared to the control ones. This decrease in Young's moduli evidences an overall softening of these structures, certainly facilitating hereby a faster migration of the growth cone ultimately leading to an increased growth velocity of neurons as observed with time lapse videomicroscopy [12]. The measured softening can be the consequence of modifications in cytoskeleton polymerization and membrane tension suggested as main drivers in the *in vitro* neurite growth [39]. Indeed, the decrease in the Young modulus corresponding to the C domain could be consistent with the increase in microtubule content and/or composition. Microtubules are heterogenous polymers in terms of posttranslational modifications, leading to acetylated or tyrosinated forms. Acetylated form of microtubules does not show colocalization with F-actin and is limited to the central region. The dynamic pool of microtubule is tyrosinated and splayed into the actin rich region and responsible for growth cone tip spreading and axon branching [18]. Immunocytochemistry data evidenced that conditioned growth cone displayed more co-localization between tubulin and actin than control growth cones, leading to less spreading of the tip. Based on these observations, it is tempting to speculate that the fast elongated mode of neurite growth is actually based on a shift towards the expression of the acetylated form of microtubules.

A way to elongate, or increase in volume, without adding cell mass is to decrease cytoplasmic density [40]. A decreased cytoplasmic density could also account for the measured increase in membrane elasticity. Consistent with a relationship between neurite growth and volume, we observed that conditioned neurons displayed larger neurite width than control neurons. This could be related to an increased volume necessary for fast elongation. Interestingly, we reported that nerve injury induced activation of the volume regulatory co-transporter Na^+^-K^+^-2Cl^−^ that could account for volume increase in neurites thereby contributing to growth velocity [12], [13]. This size increase is compatible with our observation that growth cones become much softer than those from control neurones. This might be a necessary condition to allow extension of neurite length or an effect of incorporation of structural material as previously discussed.

## Materials and Methods

### Animals and surgery

Adult female Swiss mice (6–8 weeks old CERJ, Le Genest St. Isle, France) were housed in cages with a 12 h light/dark cycle and fed and water *ad libitum*. Care and use of the mice conformed to institutional policies and guidelines. Experimental procedures were approved by the local ethics committee. All surgery was performed under isoflurane anaesthesia as previously described [12]. Briefly, the left sciatic nerve was exposed at the mid-thigh level, sectioned, and a 2–3 mm fragment of nerve was removed. To optimize the number of conditioned neurons, mice were kept alive for 4 to 5 days [11] and then killed by CO2 inhalation followed by cervical dislocation in accordance with European guidelines. Their dorsal root ganglia were then removed. For control cultures, no surgery was performed.

### Collagen-coated coverslips

For better neuron adhesion during AFM manipulations, plastic substrates were coated with collagen before neuronal culture. Collagen (Sigma Aldrich) solutions were prepared by diluting 1 mg/ml stock solution in a cold (4°C) phosphate buffer down to a concentration of 7 µg/ml. The solution's pH was then regulated to 5.8 and heated at 37°C for 15 minutes. Collagen was then absorbed on the plastic cover slips for 2 hours, and allowed to dry slowly in an oven with 95% relative humidity. This slow drying method provides a net-like patterned structure of collagen [41]. The collagen film was then coated with laminin (10 µg/ml, Sigma Aldrich) constituting the adhesion/growth factor for sensory neurons culture.

### Cell dissociation and culture

Neuronal cultures were established from either control or conditioned lumbar L4–L5 dorsal root ganglia, as previously described [11]. Ganglia were successively treated by two incubations with collagenase A (1 mg/ml, Roche Diagnostic, France) for 45 min each (at 37°C) and then with trypsin-EDTA (0.25%, Sigma, St Quentin Fallavier, France) for 30 min. They were mechanically dissociated by passing 8–10 times through the tip of a fire-polished Pasteur pipette in neurobasal (Life Technologies, Cergy Pontoise, France) medium supplemented with 10% fetal bovine serum and DNase (50 U/ml, Sigma).

Isolated cells were collected by centrifugation and suspended in neurobasal culture medium supplemented with 2% B27 (Life Technologies), 2 mM glutamine, penicillin/streptomycin (20 U/ml, 0.2 mg/ml). Dissociated neurons were plated on collagen-laminin coated plastic coverslips and were incubated in an incubator in a 95% humidified air-5% CO_2_ atmosphere. Two hours after plating, the culture medium was carefully removed and replaced to eliminate dead cells and tissue debris. The cells were maintained in culture at 37°C for one day and then used for AFM experiments. Control and conditioned culture were not performed on the same day.

### Contrast microscopy

For three dimensional live imaging, dissociated neurons were plated on glass coverslips and imaged via an optical differential interference contrast, DIC, system mounted on a Nikon TE2000-E inverted microscope equipped with a 40× objective and a thermostated sample holder.

For cell size analysis, neuronal cultures were observed with an inverted Zeiss Axiovert 200M equipped with a CCD camera (Micromax, Roper Scientific, France) and a motorized platine driven with Metamorph 7.0 software (Molecular Devices, Downingtown, USA). Phase contrast images of neurons were collected with a LD A-Plan 20× objective. Counting and measuring was performed using the ImageJ program.

### Fluorescence microscopy

For live cell height measurements, neuronal cultures at 1DIV were incubated with a fluorescent probe, CMRA (Molecular probes) at 10 µM for 60 minutes at room temperature. Following wash-out, culture was maintained at 37°C before 3D fluorescence measurements with LSM5 confocal microscope (Zeiss).

For immunocytochemistry, neuronal cultures were fixed for 15 min in 4% paraformaldehyde in PBS, and incubated for 20 min in 10% donkey serum in PBS. They were then incubated at room temperature for 2 h with the primary antibodies. Cell cultures were then incubated for 1 h at room temperature with secondary antibody, and were mounted in Mowiol. The primary antibodies used were mouse βIII-tubulin (1/500; Sigma) and rabbit anti-actin (1∶50; Sigma). The secondary antibodies used were conjugated with Alexa Fluor-594 (1∶2000) or Alexa Fluor-488 (1∶1000; Molecular Probes). Growth cone images were collected with 63× objective on LSM5 confocal Zeiss microscope.

### Atomic Force Microscopy (AFM)

The AFM experimental system used for both cell imaging and force mapping was the Asylum MFP-3D head coupled to the Molecular Force Probe 3D controller (Asylum Research, Santa Barbara,CA, USA), and mounted on an Olympus inverted microscope. Triangular silicon nitride cantilevers (MLCT-AUHW, Veeco) with a nominal spring constant of 10 pN/nm and half-opening angle of 35°, and Bio-levers (BL-RC150VB, Olympus) with a nominal spring constant of 30 pN/nm and half-opening angle of 45° were used. Prior each measurement the spring constant of cantilevers was determined using the thermal noise method within the supplied software. After one day *in vitro*, neurons were transferred under the MFP3D head of our Asylum AFM in a bathing solution containing 140 mM NaCl, 5 mM KCl, 2 mM CaCl_2_, 1.5 mM MgCl_2_, 10 mM HEPES, 10 mM glucose and the pH was adjusted to 7.4 with NaOH. This solution avoided unwanted adsorption of proteins on the AFM tip and we previously showed that it allowed recording electrical activity from control or axotomized neurons for at least 2 hours [42]. AFM measurements were never exceeding 2 hours. AFM topographic images were obtained in contact or tapping mode in liquid at an average temperature of 30°C.

**Figure 8:**
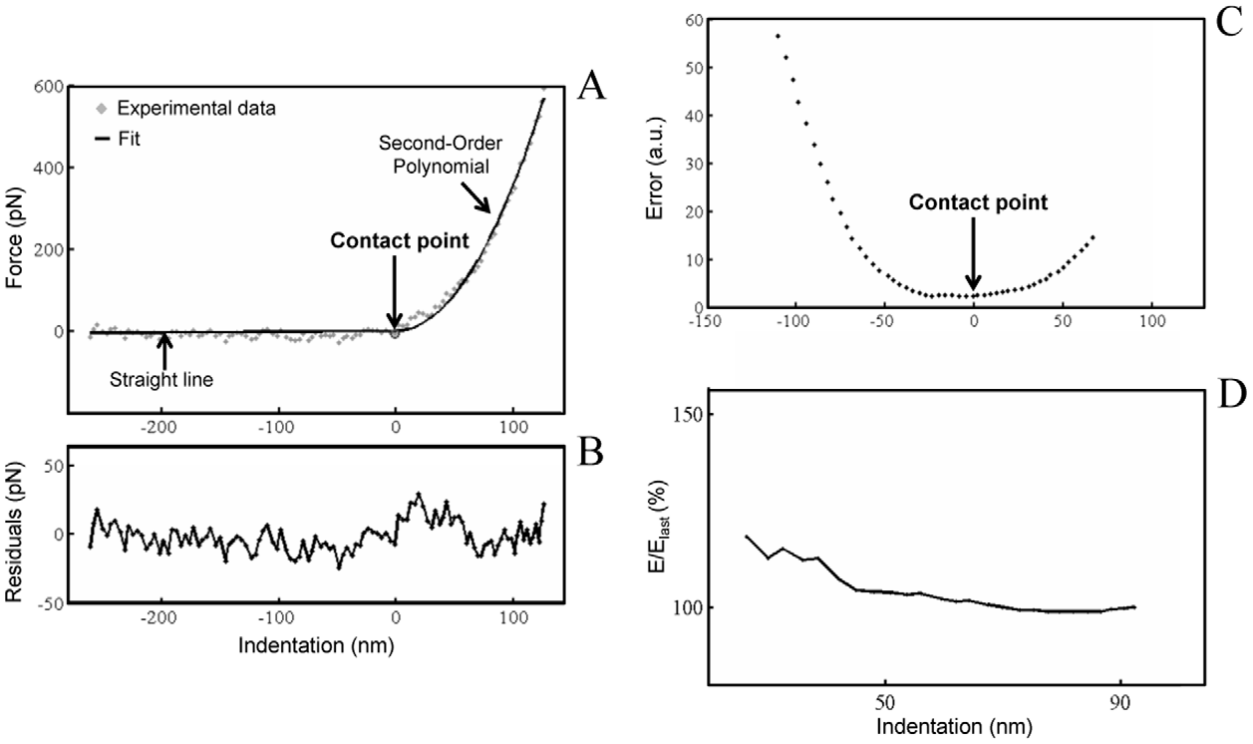
Determination of the contact point between probe and cell and dependence of the Young's modulus on indentation. (A) Approaching part of the force curve composed of two distinct processes: before tip-sample contact, it behaves as a straight line, after contact point, it behaves as a polynomial of second order. The contact point is determined where the difference between the recorded data and a theoretical curve is minimal. Evaluation at each piezo step along the vertical distance resulted in an error function which has a local minimum at the real contact point. (B) Residuals plot. (C) Error for each piezo step, calculated as a mean squared difference between the recorded data and the fitted double component curve. (D) Young's modulus is independent of indentation for indentations higher than 50 nm. Due to the point rate used to record the force curves, smaller indentations than 50 nm might lead to higher fitting errors resulting from less number of points being fitted.

Before imaging the state of the neurons was always directly examined with the inverted microscope on which is standing the MFP-3D head of our AFM, according to following previous works [30] where the health of the cells was also visually observed using the optical scope to monitor major morphological changes. In particular, this work reported that without the controlled environment of a heated stage, the neural cells began to show negative effects after 4 hours. Neurons were imaged with a pixel resolution of 512 pixels at a line rate of 0.6 Hz. During scanning both trace and retrace images were recorded and compared for accuracy. No considerable difference could be observed between them. Force curves were recorded with a tip loading speed of 6 µm/s, meaning a piezo-extension rate of 3 Hz to minimize hydrodynamic and viscoelastic artefacts [43], [44]. After testing a range of loading forces on different regions of the growth cones, the measurements were performed with a maximum loading force of about 1 nN, to get the wider fit range of the curve, and corresponding maximal indentation depth of 200–350 nm, depending on the local Young's modulus, as these loading forces yielded to the best results. Nevertheless, we also used lower loading forces values (300 pN), in order to check that forces around 1 nN did not lead to stiffness overestimation due to a possible strong influence of the substrate. We used various scan sizes and 64 or 96 points per line. From these force curves we obtain the elastic deformation as a function of the loading force applied by the tip. Then, the Young's modulus (E) was calculated for each force from the approaching part of the force curves only, as the force curves recorded for growth cones exhibited a hysteresis that in general evidences the viscoelastic behavior of the sample [45]. For each force curve the contact point between the tip and the growth cone was determined using a home made implemented Igor code added to the Asylum Research software. The approaching part of every force curve is composed of two distinct processes: before tip-sample contact, it can be described as a straight line, after reaching contact point, it behaves as a polynomial. Preliminary analysis was performed to check the order of the polynomial the curve undergoes after tip-sample contact, which has proved the behavior of second order. The contact point was determined where the difference between the recorded data and a theoretical curve is minimal. The latter was composed of a line and a second order polynomial, before and after contact point respectively (Fig. 8A, 8B). Evaluation at each piezo step along the vertical distance resulted in an error function which has a local minimum at the real contact point. The error for each piezo step was calculated as a mean squared difference between the recorded data and the fitted double component curve (Fig. 8C). The correlation coefficient of theoretical and recorded data at contact point, was mainly over R^2^ = 0.99. Smaller values than R^2^ = 0.95 were not accepted, and not included in results. Uncertainty of one step induced less than 1% error to the final Young Modulus values. For the calculation of Young's modulus we used a modified Hertz model [46] based on the work of Sneddon [47] and further developed for different AFM tip shapes [48]–[50] to fit the approaching part of the force curves, from the contact point to the maximal indentation depth. The indentation of the cantilever was calculated as a difference between the deflection obtained on a hard glass surface and those obtained on the growth cones. The loading force (F) as a function of indentation (Δz), for a conical tip with half-opening angle α is described by the equation:
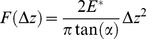
(1)Where E* is the reduced Young's modulus [51] described by:
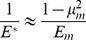
(2)E_m_ (referred to as “E” henceforth) is the Young's modulus and μ_m_ is Poisson's ratio of the growth cone, which was assumed to be 0.5 because the cell was considered incompressible [49]. Calculated Young's moduli proved to be independent of the indentation depth for indentations higher than 50 nm. In this work, all force curves have higher indentations than 100 nm, with maximal indentation depths ranging from 200 to 350 nm (Fig. 8D).

Thus, the error function used in our algorithm has a local minimum around contact point and the reduced Elastic Modulus is practically independent of indentation. Therefore, based on our estimate, the intrinsic error of reduced E due to uncertainty of contact point localization is less than 7–10% of the final value. The differences between reduced modulus values of conditioned and control growth cone values showed in the manuscript are over three fold, which is far beyond the error due to contact point uncertainty (see Fig. 6F).

### Statistical analysis

All data are expressed as the mean ± standard error of the mean (s.e.m.). Student's t-test was used for comparison between groups. A p value <0.05 was considered as significant.

## References

[1] Lawson S (1992) Morphological and biochemical cell types of sensory neurons. Sensory Neurons. Diversity, Development, and Plasticity.Oxford University Press, New York. 27–59.

[2] Araki T, Nagarajan R, Milbrandt J (2001) Identification of genes induced in peripheral nerve after injury. Expression profiling and novel gene discovery. J Biol Chem 276: 34131–34141. 11427537 10.1074/jbc.M104271200

[3] Xiao HS, Huang QH, Zhang FX, Bao L, Lu YJ, et al (2002) Identification of gene expression profile of dorsal root ganglion in the rat peripheral axotomy model of neuropathic pain. Proc Natl Acad Sci U S A 99: 8360–8365. 12060780 10.1073/pnas.122231899 PMC123072

[4] Mechaly I, Bourane S, Piquemal D, Al-Jumaily M, Venteo, et al (2006) Gene profiling during development and after a peripheral nerve traumatism reveals genes specifically induced by injury in dorsal root ganglia. Mol Cell Neurosci 32: 217–229. 16769221 10.1016/j.mcn.2006.04.004

[5] Nilsson A, Moller K, Dahlin L, Lundborg G, Kanje M (2005) Early changes in gene expression in the dorsal root ganglia after transection of the sciatic nerve; effects of amphiregulin and PAI-1 on regeneration. Brain Res Mol Brain Res 136: 65–74. 15893588 10.1016/j.molbrainres.2005.01.008

[6] Hoffman PN (2010) A conditioning lesion induces changes in gene expression and axonal transport that enhance regeneration by increasing the intrinsic growth state of axons. Exp Neurol 223: 11–18. 19766119 10.1016/j.expneurol.2009.09.006

[7] Chen ZL, Yu WM, Strickland S (2007) Peripheral regeneration. Annu Rev Neurosci 30: 209–233. 17341159 10.1146/annurev.neuro.30.051606.094337

[8] Tanaka K, Zhang QL, Webster HD (1992) Myelinated fiber regeneration after sciatic nerve crush: morphometric observations in young adult and aging mice and the effects of macrophage suppression and conditioning lesions. Exp Neurol 118: 53–61. 1397176 10.1016/0014-4886(92)90022-i

[9] Hannila SS, Filbin MT (2008) The role of cyclic AMP signaling in promoting axonal regeneration after spinal cord injury. Exp Neurol 209: 321–332. 17720160 10.1016/j.expneurol.2007.06.020 PMC2692909

[10] Smith DS, Skene JH (1997) A transcription-dependent switch controls competence of adult neurons for distinct modes of axon growth. J Neurosc 17: 646–658. 10.1523/JNEUROSCI.17-02-00646.1997 PMC6573254 8987787

[11] Andre S, Boukhaddaoui H, Campo B, Al-Jumaily M, Mayeux V, et al (2003) Axotomy-induced expression of calcium-activated chloride current in subpopulations of mouse dorsal root ganglion neurons. J Neurophysiol 90: 3764–3773. 12944538 10.1152/jn.00449.2003

[12] Pieraut S, Laurent-Matha V, Sar C, Hubert T, Mechaly I, et al (2007) NKCC1 phosphorylation stimulates neurite growth of injured adult sensory neurons. J Neurosc 27: 6751–6759. 10.1523/JNEUROSCI.1337-07.2007 PMC6672700 17581962

[13] Pieraut S, Lucas O, Sangari S, Sar C, Boudes M, et al (2011) An autocrine neuronal interleukin-6 loop mediates chloride accumulation and NKCC1 phosphorylation in axotomized sensory neurons. J Neurosc 31: 13516–13526. 10.1523/JNEUROSCI.3382-11.2011 PMC6623307 21940443

[14] Charron F, Tessier-Lavigne M (2007) The Hedgehog, TGF-beta/BMP and Wnt families of morphogens in axon guidance. Adv Exp Med Biol 621: 116–133. 18269215 10.1007/978-0-387-76715-4_9

[15] Dickson TC, Mintz CD, Benson DL, Salton SR (2002) Functional binding interaction identified between the axonal CAM L1 and members of the ERM family. J Cell Biol 157: 1105–1112. 12070130 10.1083/jcb.200111076 PMC2173555

[16] Song HJ, Poo MM (2001) The cell biology of neuronal navigation. Nat Cell Biol 3: 81–88 doi:10.1038/35060164. 10.1038/35060164 11231595

[17] Huber AB, Kolodkin AL, Ginty DD, Cloutier JF (2003) Signaling at the growth cone: Ligand-Receptor Complexes and the Control of Axon Growth and Guidance. Annual Review of Neuroscience 26: 509–563 doi:10.1146/annurev.neuro.26.010302.081139. 10.1146/annurev.neuro.26.010302.081139 12677003

[18] Dent EW, Gertler FB (2003) Cytoskeletal dynamics and transport in growth cone motility and axon guidance. Neuron 40: 209–227. 14556705 10.1016/s0896-6273(03)00633-0

[19] Costa KD (2003) Single-cell elastography: probing for disease with the atomic force microscope. Dis Markers 19: 139–154. 15096710 10.1155/2004/482680 PMC3850842

[20] Suter DM, Miller KE (2011) The emerging role of forces in axonal elongation. Prog Neurobiol 94: 91–101. 21527310 10.1016/j.pneurobio.2011.04.002 PMC3115633

[21] Xiong Y, Lee AC, Suter DM, Lee GU (2009) Topography and nanomechanics of live neuronal growth cones analyzed by atomic force microscopy. Biophys 96: 5060–5072. 10.1016/j.bpj.2009.03.032 PMC2712036 19527666

[22] Evans E, Mohandas N, Leung A (1984) Static and dynamic rigidities of normal and sickle erythrocytes. Major influence of cell hemoglobin concentration. J Clin Invest 73: 477–488. 6699172 10.1172/JCI111234 PMC425039

[23] Heinrich V, Ritchie K, Mohandas N, Evans E (2001) Elastic thickness compressibilty of the red cell membrane. Biophys J 81: 1452–1463. 11509359 10.1016/S0006-3495(01)75800-6 PMC1301624

[24] Dai J, Sheetz MP (1995) Mechanical properties of neuronal growth cone membranes studied by tether formation with laser optical tweezers. Biophys J 68: 988–996. 7756561 10.1016/S0006-3495(95)80274-2 PMC1281822

[25] Sleep J, Wilson D, Simmons R, Gratzer W (1999) Elasticity of the red cell membrane and its relation to hemolytic disorders: an optical tweezers study. Biophys J 77: 3085–3095. 10585930 10.1016/S0006-3495(99)77139-0 PMC1300579

[26] Bausch AR, Moller W, Sackmann E (1999) Measurement of local viscoelasticity and forces in living cells by magnetic tweezers. Biophys J 76: 573–579. 9876170 10.1016/S0006-3495(99)77225-5 PMC1302547

[27] Radmacher M (1997) Measuring the elastic properties of biological samples with the AFM. IEEE Eng Med Biol Mag 16: 47–57. 10.1109/51.582176 9086372

[28] Mathur AB, Collinsworth AM, Reichert WM, Kraus WE, Truskey GA (2001) Endothelial, cardiac muscle and skeletal muscle exhibit different viscous and elastic properties as determined by atomic force microscopy. J Biomech 34: 1545–1553. 11716856 10.1016/s0021-9290(01)00149-x

[29] Chumakova OV, Liopo AV, Chizhik SA, Tayurskaya VV, Gerashchenko LL, et al (2000) Effects of ethanol and acetaldehyde on isolated nerve ending membranes: study by atomic-forced microscopy. Bull Exp Biol Med 130: 921–924. 11177282

[30] Mustata M, Ritchie K, McNally HA (2010) Neuronal elasticity as measured by atomic force microscopy. J Neurosci Meth 186: 35–41. 10.1016/j.jneumeth.2009.10.021 19896979

[31] Lal R, Drake B, Blumberg D, Saner DR, Hansma PK, et al (1995) Imaging real-time neurite outgrowth and cytoskeletal reorganization with an atomic force microscope. Am J Physiol 269: 275–285. 10.1152/ajpcell.1995.269.1.C275 7631755

[32] Tojima T, Yamane Y, Takagi H, Takeshita T, Sugiyama T, et al (2000) Three-dimensional characterization of interior structures of exocytotic apertures of nerve cells using atomic force microscopy. Neuroscience 101: 471–481. 11074169 10.1016/s0306-4522(00)00320-1

[33] McNally HA, Borgens RB (2004) Three-dimensional imaging of living and dying neurons with atomic force microscopy. J Neurocytol 33: 251–8. 15322383 10.1023/b:neur.0000030700.48612.0b

[34] McNally HA, Rajwa B, Sturgis J, Robinson JP (2005) Comparative three-dimensional imaging of living neurons with confocal and atomic force microscopy. J Neurosci Methods 142: 177–184. 15698657 10.1016/j.jneumeth.2004.08.018

[35] Spedden E, White JD, Naumova EN, Kaplan DL, Staii C (2012) Elasticity maps of living neurons measured by combined fluorescence and atomic force microscopy. Biophys J 113: 868–877. 10.1016/j.bpj.2012.08.005 PMC3433610 23009836

[36] García R, Magerle R, Perez R (2007) Nanoscale compositional mapping with gentle forces. Nat Mater 6: 405–411. 17541439 10.1038/nmat1925

[37] Lankford K L, Waxman S G, Kocsis J D (1998) Mechanisms of Enhancement of Neurite Regeneration In Vitro Following a Conditioning Sciatic Nerve Lesion. J Comp Neurol 391 (1) 11–29. 9527536 10.1002/(sici)1096-9861(19980202)391:1<11::aid-cne2>3.0.co;2-u PMC2605358

[38] Laishram J, Kondra S, Avossa D, Migliorini E, Lazzarino M, et al (2009) A morphological analysis of growth cones of DRG neurons combining atomic force and confocal microscopy. J Struct Biol 168: 366–77. 19747551 10.1016/j.jsb.2009.09.005

[39] Geiger B, Spatz JP, Bershadsky AD (2009) Environmental sensing through focal adhesions. Nat Rev Mol Cell Biol 10: 21–33. 19197329 10.1038/nrm2593

[40] Goldberg JL (2008) How does an axon grow? Genes Dev 17: 941–58. 10.1101/gad.1062303 12704078

[41] Dupont-Gillain ChC, Pamula E, Denis FA, De Cupere VM, Dufrene YF, et al (2004) Controlling the supramolecular organisation of adsorbed collagen layers. J Mater Sci Mater Med 15 (4) 347–53. 15332598 10.1023/b:jmsm.0000021100.71256.29

[42] Hilaire C, Inquimbert P, Al-Jumaily M, Greuet D, Valmier J, et al (2005) Calcium dependence of axotomized sensory neurons excitability. Neurosci Lett 380: 330–334. 15862912 10.1016/j.neulet.2005.01.068

[43] Rosenbluth MJ, Lam WA, Fletcher DA (2006) Force microscopy of nonadherent cells: A comparison of leukimia cell deformability. Biophys J 90: 2994–3003. 16443660 10.1529/biophysj.105.067496 PMC1414579

[44] Radmacher M, Fritz M, Kacher CM, Clevelant JP, Hansma PK (1996) Measuring the viscoelastic properties of human platelets with the atomic force microscope. Biophys J 70: 556–567. 8770233 10.1016/S0006-3495(96)79602-9 PMC1224955

[45] Lanero TS, Cavalleri O, Krol S, Rolandi R, Gliozzi A (2006) Mechanical properties of single living cells encapsulated in polyelectrolyte matrixes. J Biotechnol 124: 723–31. 16600412 10.1016/j.jbiotec.2006.02.016

[46] Hertz MG (1881) Uber die Beruhrung Fester Elastischer Korper. J Reine Angew Math 92: 156–171.

[47] Sneddon IN (1965) The relation between load and penetration in axisymmetric Boussinesq problem for a punch of arbitrary profile. Int J Engr Sci 3: 47–57.

[48] Weisenhorn AL, Khorsandi M, Kasas S, Gotzos V, Butt HJ (1993) Deformation and height anomaly of soft surfaces studied with an AFM. Nanotechnology 4: 106–113.

[49] Vinckier A, Semenza G (1998) Measuring elasticity of biological materials by atomic force microscopy. FEBS Lett 430: 12–16. 9678586 10.1016/s0014-5793(98)00592-4

[50] Butt H-J, Cappella B, Kappl M (2005) Force measurements with the atomic force microscope: Technique, interpretation and applications. Surf Sci Reports 59: 1–152 (2005).

[51] Butt H-J, Kappl M (2010) Surface and interfacial Forces. WILEY-VCH Verlag GmbH & Co. KGaA, Weinheim.

